# Ectopic Expression of *AhDef1* Gene (Peanut Defensin Gene) Reveals Enhanced Fungal Resistance and Aflatoxin Suppression in Peanut (*Arachis hypogaea* L.)

**DOI:** 10.3390/cimb48090858

**Published:** 2026-08-24

**Authors:** Nirmala Kumari Gupta, Jaykumar Patel, Gohil Mrunaliniba Yuvrajsinh, Saif Syed, Deepesh Khandwal, Avinash Mishra

**Affiliations:** 1 Division of Applied Phycology and Biotechnology, CSIR-Central Salt and Marine Chemicals Research Institute, Bhavnagar 364002, India; 2 Academy of Scientific and Innovative Research (AcSIR), Ghaziabad 201002, India

**Keywords:** aflatoxin, genetic transformation, groundnut, peanut, transgenic

## Abstract

The aim of the present study is functional characterization of a native plant defensin gene (*AhDef1*) from *Arachis hypogaea* (peanut). Further its potential has been evaluated to enhance resistance against *Aspergillus flavus* infection and reduce aflatoxin accumulation through transgenic intervention. Transgenic peanut lines overexpressing the *AhDef1* gene exhibited significantly improved resistance to *A. flavus* colonization and a marked reduction in aflatoxin B_1_ content compared to wild-type (WT) plants. Quantitative real-time PCR confirmed transgene expression, and aflatoxin accumulation was analyzed by a spectrophotometer which revealed a reduction in aflatoxin levels in the transgenic seeds. Alongside its direct antifungal activity, *AhDef1* overexpression also triggered the upregulation of genes which are involved in the biosynthesis of secondary metabolites such as resveratrol, ferulic acid and butenedioic acid, myoinositol, octadecanoic acid, suggesting an amplified biochemical defense response. Multivariate analysis further suggested that accumulation of these defense-related metabolites was positively correlated with transgenic lines challenged by *A. flavus*. In conclusion, the *AhDef1* gene functionally validated in this study emerges as a promising candidate for engineering fungal disease resistance and aflatoxin mitigation in peanut and potentially other susceptible crops.

## 1. Introduction

Peanut (*Arachis hypogaea* L.) is an economically significant legume crop cultivated extensively for its edible seeds, which are rich in oil and protein. However, its cultivation is frequently challenged by fungal pathogens, especially *Aspergillus flavus*, which infects seeds and leads to the production of aflatoxins—highly toxic and carcinogenic secondary metabolites, which can significantly reduce yield and quality, necessitating the development of improved cultivars through molecular and genetic approaches [1,2,3]. Aflatoxin contamination poses a major threat to food safety and international trade, prompting an urgent need for effective control strategies. Gene cloning and functional characterization have emerged as essential strategies for understanding key regulatory genes involved in stress responses, growth, and developmental pathways in plants [4].

Recent advances in genomics and transcriptomics have facilitated the identification of candidate genes with potential agronomic benefits. However, the functional validation of such genes, especially in peanut, remains limited due to the complexity of its tetraploid genome and relatively slow transformation process [5,6]. In this context, identifying and overexpressing native peanut genes provides a promising route for crop improvement while maintaining genetic compatibility.

Recent transcriptomic studies have identified numerous stress-responsive genes in peanut, providing a valuable resource for gene discovery and functional characterization [6]. Functional validation of native peanut genes through transgenic approaches has further demonstrated the importance of elucidating gene function for improving stress tolerance and crop performance [7]. Moreover, genome-wide analysis of the peanut defensin gene family has revealed that defensins constitute an important class of cysteine-rich antimicrobial peptides involved in plant immunity, with several members contributing to disease resistance [8]. Despite these advances, the functional role of native peanut defensins in resistance to *Aspergillus flavus* infection and aflatoxin B_1_ accumulation remains poorly understood. Therefore, *AhDef1*, a native peanut defensin gene, was selected for functional characterization to investigate its potential role in enhancing fungal resistance and mitigating aflatoxin contamination in peanut.

Although several plant defensins have been reported to enhance resistance against fungal pathogens in heterologous plant systems, functional characterization of native peanut defensin genes remains limited, particularly with respect to their role in restricting *Aspergillus flavus* colonization and reducing aflatoxin accumulation [9,10,11]. Furthermore, little information is available on the metabolic changes associated with defensin-mediated resistance in peanut [12,13,14]. Consequently, it remains unclear whether overexpression of a native peanut defensin can simultaneously enhance antifungal defense, suppress aflatoxin biosynthesis, and influence defense-related metabolic responses during fungal infection. Based on this knowledge gap, the present study aimed to functionally characterize the native peanut defensin gene *AhDef1* through *Agrobacterium*-mediated genetic transformation.

In the present study, we report the isolation, cloning, and molecular characterization of a *defensin* gene from peanut, herein referred to as *AhDef1*. Defensins are small, cysteine-rich antimicrobial peptides that play a crucial role in the innate immune system of plants [2,8]. They act as the first line of defense against a broad spectrum of pathogens, particularly fungi, by disrupting pathogen cell membranes or interfering with essential cellular processes [15]. Plant defensins have been well characterized in several species, and their overexpression has been shown to enhance resistance to fungal infections [10].

Based on the current knowledge gap, we hypothesized that constitutive overexpression of the native peanut defensin gene *AhDef1* would enhance resistance to *Aspergillus flavus*, reduce aflatoxin B_1_ accumulation, and be associated with changes in defense-related metabolites. Therefore, the objectives of this study were to (i) clone and functionally characterize the *AhDef1* gene, (ii) generate transgenic peanut plants overexpressing *AhDef1*, (iii) evaluate their resistance to *A. flavus* infection and aflatoxin B_1_ accumulation, and (iv) investigate changes in polyphenol composition and metabolite profiles using HPLC and GC–MS analyses. This work provides new insights into the potential application of defensin genes in engineering peanut varieties with improved resistance to fungal infection and reduced aflatoxin contamination, offering a promising strategy for enhancing crop safety and quality.

## 2. Materials and Methods

### 2.1. Isolation and Cloning of the Defensin Gene (AhDef1)

The total RNA was extracted from a peanut plant sample using the RNeasy Plant Mini kit (Qiagen, Hilden, Germany) according to the manufacturer’s instructions, and cDNA was synthesized using the QuantiTect Rev.Transcription Kit (Qiagen, Hilden, Germany). The full-length coding sequence of the defensin gene (*AhDef1*) was amplified using gene-specific primers designed based on conserved sequences of plant defensins (Supplementary Table S1). The PCR product was cloned into the *pGEM-T* Easy entry vector (Promega, Madison, WI, USA) and sequenced to confirm identity.

### 2.2. Vector Construction and Agrobacterium-Mediated Transformation

The confirmed recombinant plasmid *AhDef1*: pGEMT was subcloned into the vector *pRT101* under the control of the CaMV 35S promoter, replacing the GUS gene. Then the gene expression cassette containing 35S: *AhDef1*: poly A was introduced into pCAMBIA 1301. Ligated pCAMBIA1301: 35S: *AhDef1*: poly A was transformed in *E. coli* DH5α cells. The plant expression vector construct pCAMBIA1301: *AhDef1* was mobilized into the *Agrobacterium tumefaciens* strain (EHA105) by the freeze–thaw method.

### 2.3. Plant Material and Growth Conditions

Seeds of a widely cultivated peanut variety (*Arachis hypogaea* L., [GG-20]) were used for gene isolation and genetic transformation. Plants were grown in a controlled condition under standard conditions (28 ± 2 °C, 16/8 h light/dark photoperiod, and ~60% relative humidity).

### 2.4. Genetic Transformation of Peanut

Dry and mature seeds of the groundnut cultivar GG-20 were surface-sterilized with ethanol and 0.1% HgCl_2_ and then rinsed and soaked for 3 h. Seed coats and embryo axes were removed to obtain de-embryonated cotyledons. These were vertically halved and used as explants for regeneration and transformation [16,17]. Groundnut regeneration was carried out using MS salts with B5 vitamins, 3% sucrose, and 0.8% agar (pH 5.8), supplemented with different phytohormone combinations. The de-embryonated cotyledon explants underwent direct organogenesis using shoot induction media and shoot elongation media (EM1–EM3) protocols, compared for regeneration efficiency and shoot bud induction [16,17]. The cultures were maintained at 25 ± 2 °C under a 16/8 h photoperiod with 60 μmol m^−2^ s^−1^ light intensity. Elongated shoots of approximately 2–3 cm, with well-developed stems, were sub-cultured in the rooting media. The rooting media were designated as rooting media. After 2–3 rounds of sub-culturing, rooted plantlets were transferred to Soilrite, acclimatized under polyethylene covers for 2 weeks, and then moved to greenhouse conditions [17].

### 2.5. Confirmation of Transgene Integration

Transgene integration in groundnut was confirmed by PCR using genomic DNA isolated from leaves of T_0_ transgenic and wild-type (WT) plants [17]. PCR was performed using *hptII*-specific primers (hptF-hptR) in a 25 µL reaction containing buffer, 1.5 mM MgCl_2_, 0.2 mM dNTPs, 5 pmol primers, 1.25 U Taq polymerase, and 200 ng DNA. Amplicons were resolved on 1.0% agarose gel, stained with ethidium bromide, and visualized using a Bio-Rad Gel Doc system. Putative transgenic shoots were selected on Murashige and Skoog (MS) medium supplemented with 30 mg/L hygromycin for the selection of transformed tissues. Hygromycin-tolerant plants were further conferred by PCR using the *hptII*-specific primers.

### 2.6. Copy Number Detection

PCR-confirmed lines were subsequently evaluated for copy number analysis via qRT-PCR using designated primers. For that, gDNA was extracted from selected transgenic plants leaves, adhering to the protocol of the DNeasy Plant Mini kit (Qiagen, Hilden, Germany). Agarose gel electrophoresis and a ND-8000 spectrophotometer (Thermo Scientific NanoDrop, Waltham, MA, USA) were employed to ascertain the quantity and integrity of the gDNA. To prepare for further analysis, serial dilutions were conducted on the genomic DNA, yielding different concentrations including 100, 10, 1, 0.1, and 0.01 ng µl^−1^. This serially diluted genomic DNA was then used as a template for qRT-PCR. For the copy number analysis, primers (Supplementary Table S1) specific to the single-copy gene nitrate reductase (NRA) and *uidA* were utilized. The analysis was carried out as per the given program (Supplementary Table S2). The *defensin* gene was used as the target, while vacuolar protein sorting-associated protein53 A-like (GnVP) was used as the internal reference gene. The copy number of the reference gene for GnVp (Gene ID: 107638771) was taken as 2 copies in the tetraploid groundnut for copy number estimation [2].

### 2.7. Transgene Expression Analysis in the Transgenic Lines

The total RNA was isolated from leaf tissues of transgenic lines and WT plants using the RNeasy Plant Mini Kit (Qiagen, Germany). The cDNA was prepared using a reverse transcriptase (PrimeScript™ 1st strand cDNA Synthesis Kit, Takara, Kyoto, Japan) and used for RT-qPCR containing 100 ng cDNA. RT-qPCR reactions were performed [6] in a CFX96™ Real-Time PCR Detection System (Bio-Rad, Hercules, CA, USA) using TAKARA TB Green Premix Ex Taq II (Tli RNase H Plus). Each 25 µL reaction contained 12.5 µL of TB Green Premix Ex Taq II (Tli RNaseH Plus) (2X), 10 µM each of forward and reverse primers, 1 µL of cDNA template (diluted 1:10), and nuclease-free water. The amplification program consisted of an initial denaturation at 95 °C for 3 min, followed by 40 cycles of 95 °C for 15 s and 60 °C for 30 s. Melt curve analysis was performed from 65 to 95 °C at 0.5 °C increments to confirm specificity. Here the housekeeping gene *actin* was taken as a positive control. Relative transcript abundance was calculated using the 2^^−ΔΔCt^ method [18].

### 2.8. Aspergillus flavus Growth Conditions

The virulent strain *Aspergillus flavus*, originally obtained from the groundnut pathology collection at ICRISAT (Hyderabad, India), was used for fungal bioassays [3]. The cultures were maintained on potato dextrose agar (PDA) at 28 °C until profuse sporulation was observed. Conidial suspensions were prepared by flooding sporulating cultures with sterile distilled water supplemented with 0.05% (*v*/*v*) Tween-20, followed by filtration through sterile muslin cloth to remove hyphal fragments. Spore concentrations were determined using a Neubauer hemocytometer and adjusted to 5 × 10^4^ conidia mL^−1^. Colony-forming units (CFUs) were verified by standard 10-fold serial dilution plating on *A. flavus parasiticus* agar (AFPA), yielding ~4 × 10^4^ CFU mL^−1^ [3,19].

### 2.9. Fungal Inoculation and Aflatoxin B1 Quantification

Mature seeds from T1 transgenic and non-transgenic (wild-type) peanut plants were surface-sterilized and inoculated with a toxigenic strain of *Aspergillus flavus* (AF 11-4). Seeds were incubated at 28 °C with high humidity [19]. Aflatoxin B1 levels in mature seeds from both transgenic (*AhDef1*-overexpressing) and wild-type peanut plants were quantified using a competitive enzyme-linked immunosorbent assay (ELISA) kit specific for AFB1 (RIDASCREEN Aflatoxin B1 30/15 Art. No. R1211). The assay was performed according to the manufacturer’s protocol and as described by Atasever et al. [20]. Briefly, 100 mg of grounded seed material was extracted in 70% methanol and filtered. A 100 µL aliquot of the filtrate was subjected to ELISA following kit instructions. Absorbance was measured at 450 nm using a microplate reader, and quantification was performed using a six-point calibration curve consisting of standards at 0, 1, 5, 10, 20, and 50 µg/L, following the manufacturer’s instructions. AFB_1_ concentrations were determined by interpolation from a four-parameter logistic (4PL) standard curve constructed from the ELISA standards. The interpolated concentrations (µg/L) were converted to seed concentrations (µg/kg) by accounting for the extraction volume and sample weight [21].

### 2.10. Polyphenol Quantification

Polyphenols were extracted from peanut seed samples following a modified methanolic extraction protocol. Briefly, 1 g of finely ground seed powder was mixed with 10 mL of 80% methanol (*v*/*v*) and sonicated for 20 min at room temperature. The mixture was centrifuged at 10,000× *g* for 15 min at 4 °C, and the supernatant was collected. The extraction was repeated twice, and supernatants were pooled and filtered through a 0.22 µm PTFE syringe filter (Millipore, Burlington, MA, USA) before analysis.

Polyphenol quantification was performed using a high-performance liquid chromatography (HPLC) system (Shimadzu LC-20AT, Kyoto, Japan) equipped with a reversed-phase C18 column (Phenomenex Luna, 250 × 4.6 mm, 5 µm particle size). The mobile phase consisted of solvent A (0.1% formic acid in water) and solvent B (acetonitrile) under a gradient elution program: 0–5 min, 5% B; 5–25 min, 5–40% B; 25–35 min, 40–70% B; 35–40 min, 70–5% B, at a flow rate of 1 mL/min. The injection volume was 40 µL, and detection was performed at 280 nm using a UV–Vis detector. Quantification of individual polyphenols was carried out using calibration curves prepared with authentic standards (e.g., caffeic acid, ferulic acid, resveratrol). The results were expressed as µg/g of dry weight.

### 2.11. Metabolomics of Transgenic Plants

Metabolites were extracted and quantified using an optimized GC–MS protocol [3]. Briefly, 100 mg of dried, powdered peanut seed samples from transgenic and wild-type plants (48, 72, and 96 h post-*Aspergillus flavus* infection) were extracted with cold methanol by vertexing, followed by incubation at 70 °C for 10 min and centrifugation at 11,000× *g* for 10 min at 4 °C. The resulting supernatant was collected and subjected to a second extraction with chloroform. The upper phase was aspirated, dried, and derivatized. For metabolite quantification, 6 µg of ribitol was added as an internal standard. Derivatization was carried out by adding 40 µL of methoxyamine hydrochloride (in pyridine) and 70 µL of N-methyl-N-(trimethylsilyl) trifluoroacetamide (MSTFA). The derivatized samples were analyzed using a gas chromatography–mass spectrometry system (GC–MS, Shimadzu, Kyoto, Japan). Mass spectra were recorded, and metabolites were identified by comparing spectral peaks against the NIST mass spectral library. Metabolomic data were analyzed using MetaboAnalyst ver. 6.0 [22]. Metabolite concentrations were median-normalized to minimize technical variation, log_10_-transformed to improve data distribution, and auto-scaled (mean-centered and divided by the standard deviation) prior to metabolic network analysis.

The study consisted of wild-type (WT) and *AhDef1*-overexpressing transgenic peanut lines. Seeds from both groups were artificially inoculated with *Aspergillus flavus* and sampled at 48, 72, and 96 h after inoculation. Non-inoculated seeds served as controls where appropriate. A1S, A2S, and A3S represent *AhDef1*-overexpressing transgenic peanut seed samples collected at 48, 72, and 96 h, respectively, following *Aspergillus flavus* inoculation. Similarly, B1S, B2S, and B3S represent wild-type peanut seed samples collected at the corresponding time points (48, 72, and 96 h) after inoculation.

### 2.12. Statistical Analysis

The data represent the mean of three independent replicates, and the error bars represent standard deviation. Statistically significant differences were calculated by ANOVA (Tukey’s HSD) test and shown by different letters associated with error bars (*p*-value ≤ 0.05).

## 3. Results

### 3.1. Structural Prediction and Topology Analysis of AhDef1 Protein

The in silico secondary structure and topology predictions revealed that the *Ah*Def1 protein possesses a compact, membrane-associated conformation typical of plant defensins (Supplementary Figure S1). The DeepConCNF SS8 prediction indicated that *Ah*Def1 contains two prominent α-helical regions (residues 4–30 and 48–54), separated by a short flexible coil (residues 31–37) and followed by a β-strand (residues 63–71). This organization forms a characteristic helix–loop–helix–β motif, commonly observed in cysteine-rich antimicrobial peptides. The N- and C-terminal regions were predicted to be coil-dominated, reflecting conformational flexibility that may facilitate interaction with membranes or target proteins.

The SS3 probability profile provided additional confidence for this architecture, displaying a high α-helix probability (>0.9) in the N-terminal region and moderate β-sheet probability toward the C-terminus. The coexistence of α-helices and β-strands suggests a compact, stable tertiary fold stabilized by disulfide linkages—a hallmark of plant defensins. These disulfide bridges are predicted to link conserved cysteine residues, maintaining structural rigidity under stress conditions.

Complementary Protter topology mapping predicted a single transmembrane α-helix spanning residues approximately 10–30, confirming that *Ah*Def1 is a single-pass membrane-anchored protein. The N-terminal domain is oriented toward the extracellular side, where a putative N-linked glycosylation site (Asn) was identified, while the C-terminal cysteine-rich domain is positioned extracellularly. This arrangement is consistent with the architecture of membrane-anchored defensin-like proteins that function in pathogen recognition and stress signaling. The hydrophobic α-helical core likely serves as a membrane anchor, while the C-terminal domain, stabilized by multiple cysteine residues, is predicted to mediate extracellular antimicrobial activity or signaling interactions.

Collectively, these analyses indicate that *Ah*Def1 adopts a single-pass transmembrane topology with a stable α-helical core and a disulfide-stabilized extracellular tail. Such a configuration is characteristic of plant defensins involved in membrane stabilization, pathogen inhibition, and signal transduction, reinforcing the functional role of *Ah*Def1 in defense and stress tolerance mechanisms.

### 3.2. Cloning of Defensin Gene in Plant Expression (pRT101) and Plant Transformation (pCAMBIA1301) Vector

The full-length coding sequence of *AhDef1* was successfully amplified (Supplementary Figure S2) from peanut cDNA using gene-specific primers, yielding a product of the expected size (~317 bp) as confirmed by agarose gel electrophoresis (Supplementary Figure S3). The amplicon was cloned into the *pGEM-T* Easy vector, and Sanger sequencing confirmed 100% identity with predicted peanut defensin gene sequences in the database. The verified *AhDef1* insert was subcloned into the plant expression vector *pRT101* under the CaMV35S promoter, replacing the GUS reporter gene. Restriction digestion and PCR analyses confirmed correct orientation and integrity of the cloned insert (Supplementary Figure S3). The expression cassette (35S:*AhDef1*:polyA) was further mobilized into the binary vector pCAMBIA1301. Positive *E. coli* DH5α colonies carrying the recombinant construct were verified by colony PCR and double digestion (Supplementary Figure S3). Finally, the binary vector *pCAMBIA1301:AhDef1* was successfully introduced into *Agrobacterium tumefaciens* strain EHA105 by the freeze–thaw method. Recombinant *Agrobacterium* colonies were confirmed by colony PCR, demonstrating the presence of the defensin insert (Supplementary Figure S3). These validated constructs were subsequently used for peanut transformation.

### 3.3. Genetic Transformation and Regeneration of Transgenic Plants

**Figure 1:**
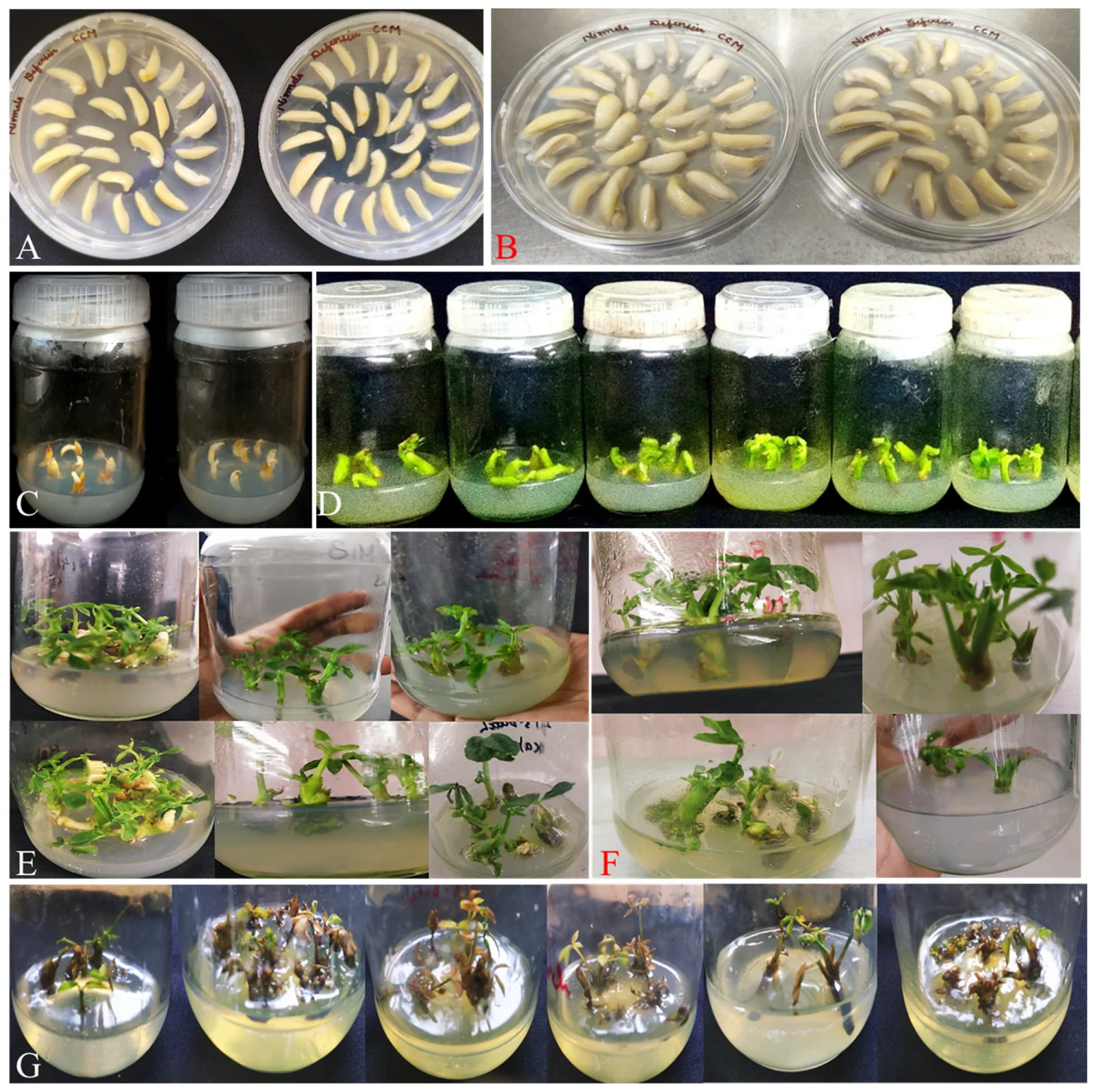
Tissue culture and genetic transformation in peanut. (**A**) Explants on cocultivation media (Day 1); (**B**) explants on cocultivation media (Day 5); (**C**) transformants transferred to shoot induction medium (SIM) after 5 days; (**D**) transformants on shoot elongation medium (SEM) after 15 days supplemented with 15 mg/L hygromycin; (**E**) transformants on SEM after 30 days supplemented with 30 mg/L hygromycin; (**F**) transformants on rooting media after 15 days; and (**G**) untransformed shoots on 30 mg/L hygromycin.

**Figure 2:**
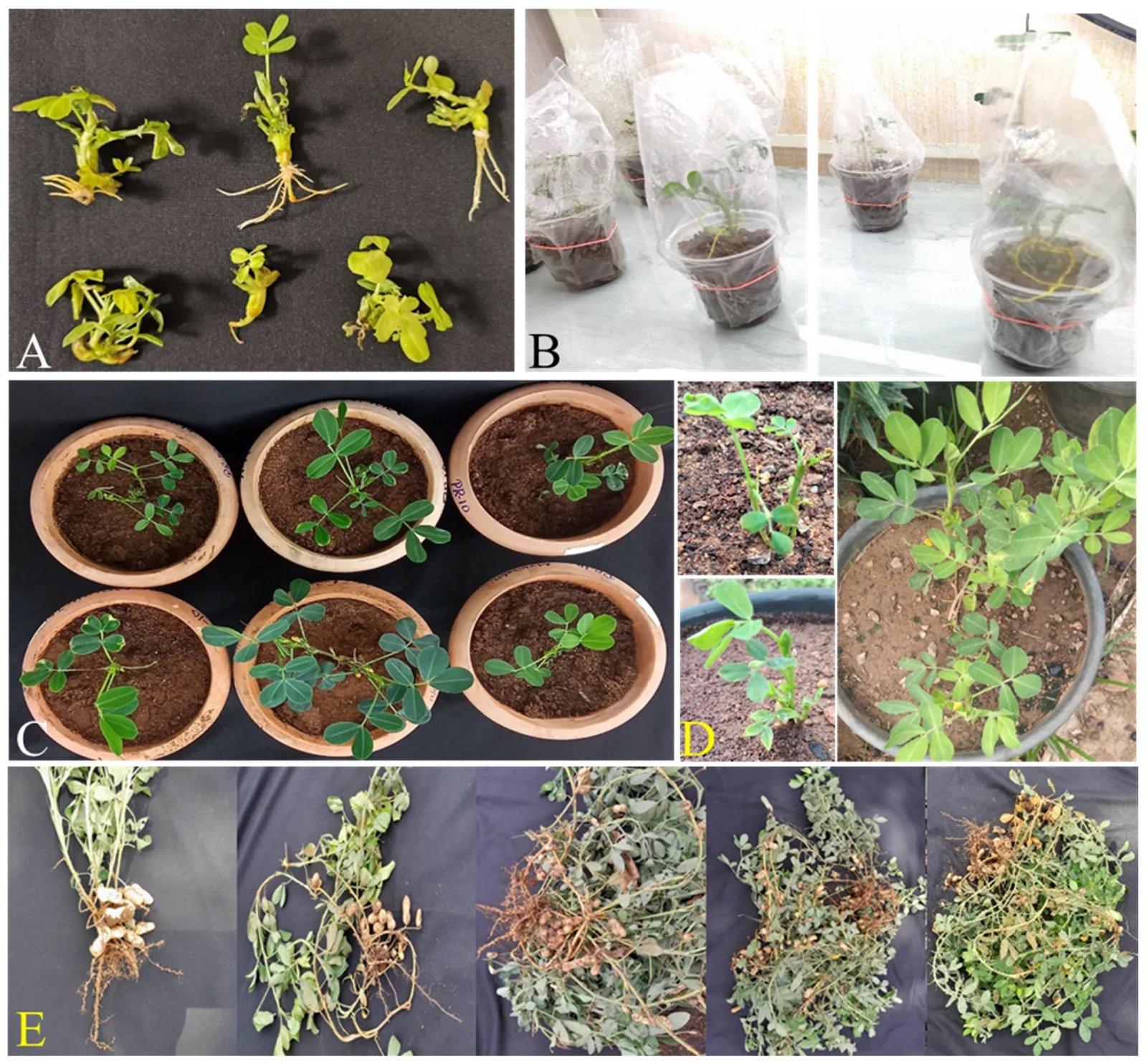
Development of transgenic peanut. (**A**) Putative transgenic plants ready to be potted in soil; (**B**) acclimatization of putative transgenic plants; (**C**) one-month-old putative transgenic plants in pot (soil); (**D**) three-month-old putative transgenic peanut plants; and (**E**) harvested putative transgenic peanut plants with seeds.

Following *Agrobacterium*-mediated transformation, infected explants were cultured on co-cultivation medium (CCM). After three successive rounds of selection on shoot induction medium (SIM) and shoot elongation medium (SEM), only transformed explants survived, while non-transformed tissues exhibited necrosis and failed to regenerate beyond the second subculture. Hygromycin-resistant shoots were subsequently transferred to rooting medium, where stable root induction was achieved (Figure 1). Putative transformants were acclimatized under controlled laboratory conditions and subjected to molecular confirmation. Verified transgenic lines were then established in the greenhouse, grown to maturity, and advanced to seed production for downstream analyses (Figure 2).

Approximately 100 de-embryonated cotyledon explants were subjected to *Agrobacterium tumefaciens*-mediated transformation using the *AhDef1* expression construct in different experiments. Each regenerated transgenic plant represented an independent transformation event arising from a separate transformed explant. Following selection, regeneration, rooting, acclimatization, and greenhouse establishment, five independent transgenic plants successfully reached maturity and produced seeds. These plants were screened by PCR and transgene copy number analysis, and two independent single-copy transgenic lines were selected for subsequent gene expression, fungal infection and biochemical analyses.

### 3.4. Molecular Characterization of Transgenic Plants

Acclimatized putative transgenic lines were confirmed for stable integration of the transgene into the groundnut genome by PCR with *hptII* primers and defensin primers (Supplementary Figure S4).

### 3.5. Copy Number Analysis

The number of transgene copies integrated into the host genome is a critical parameter when evaluating gene expression in transgenic peanut lines. A higher copy number may result in increased transgene expression, potentially affecting the interpretation of downstream analyses. Therefore, the transgene copy number was determined for the defensin gene in the transformed peanut lines. Quantitative real-time PCR (qRT-PCR) was employed to estimate the copy number in T_1_ transgenic plants. Serial dilutions of genomic DNA from 100 ng to 0.1 ng were used to prepare the standard curves. A defensin: GnVp ratio between 0.5 and 1.5 was considered indicative of a single-copy integration of the transgene cassette in the genome. Based on this analysis, two independent single-copy transgenic lines were identified and selected for further expression and metabolomic studies (Supplementary Table S2).

### 3.6. Defensin Gene Expression Analysis

**Figure 3:**
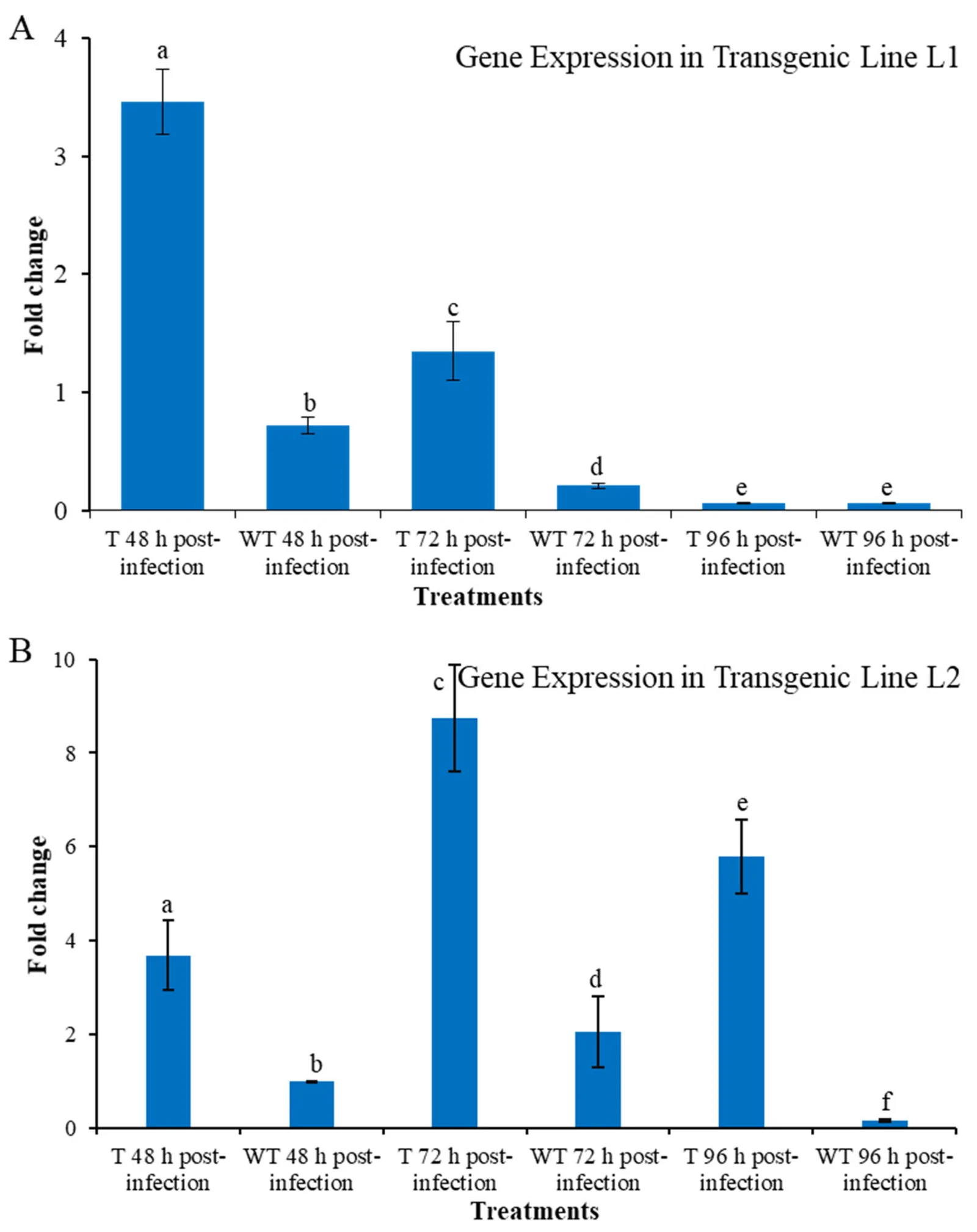
Gene (*AhDef1*) expression analysis in wild-type and transgenic peanut seeds after *A. flavus* infection. (**A**) Transgenic line 1 and (**B**) transgenic line 2. Data are mean ± SE (*n* = 3), and different letters show statistically significant differences (*p*-value ≤ 0.05).

Gene expression analysis was performed using two independent single-copy transgenic lines and wild-type plants. For each treatment, three biological replicates were analyzed, and each biological replicate was subjected to three technical qRT-PCR replicates. The expression pattern of the defensin transgene in transgenic peanut lines was evaluated by quantitative real-time PCR (qRT-PCR) across different time points following *Aspergillus flavus* infection (Figure 3). Transcript levels were normalized to actin as an internal reference, and relative expression was calculated. For transgenic line 1, a substantial induction of defensin expression was observed at 48 h post-infection, showing a 3.4-fold increase relative to the 0 h control. In contrast, wild-type plants exhibited a lower expression level at the corresponding time point (0.72-fold). At 72 h, transgenic lines maintained elevated expression (1.3-fold), whereas wild-type plants showed a marked decline to 0.2-fold relative to the baseline. These results confirm stable expression and pathogen-responsive induction of the defensin transgene in peanut, with peak transcription observed at 48 h post-infection (Figure 3). For transgenic line 2, the defensin transcript was strongly induced at 48 h post-infection, exhibiting a 3.5-fold increase compared with the 0 h baseline, while wild-type plants showed no induction at the same time point. Notably, defensin expression peaked at 72 h post-infection, reaching an 8.6-fold increase relative to baseline, whereas wild-type plants displayed only a modest 1.8-fold increase (Figure 3). This pronounced upregulation of defensin expression in transgenic lines indicates a robust and time-dependent transcriptional response to fungal challenge.

**Figure 4:**
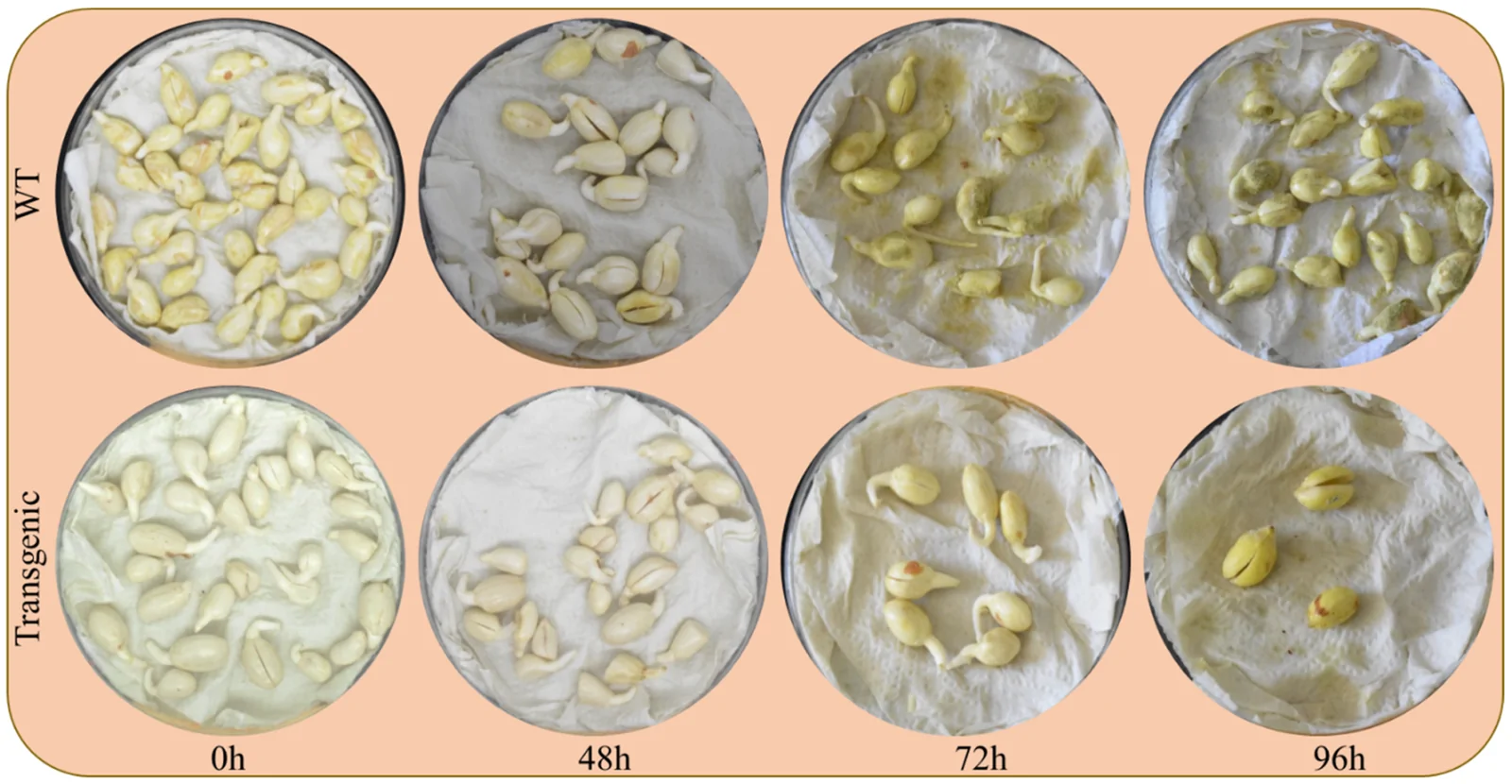
*A. flavus* infection in wild-type and transgenic peanut seeds.

Upon artificial inoculation with the *A. flavus* strain, clear differences in infection progression were observed between wild-type (WT) and defensin-expressing transgenic (T) seeds (Figure 4). At 0 h post-inoculation, both WT and T seeds appeared healthy and free of visible fungal colonization. By 48 h, WT seeds exhibited initial signs of infection, including tissue softening and mild surface discoloration, whereas transgenic seeds remained largely unaffected, maintaining intact morphology. At 72 h, WT seeds showed extensive fungal colonization characterized by greenish mycelial growth and sporulation, while transgenic seeds displayed only minor discoloration with negligible fungal biomass. By 96 h, WT seeds were heavily colonized with dense fungal growth covering the seed surface, in contrast to the transgenic seeds, which showed only minimal infection and retained structural integrity. These observations demonstrate that expression of the peanut defensin gene significantly restricts fungal colonization and delays disease progression under the *A. flavus* challenge (Figure 4).

### 3.7. Reduced Aflatoxin Accumulation in Transgenic Seeds

**Figure 5:**
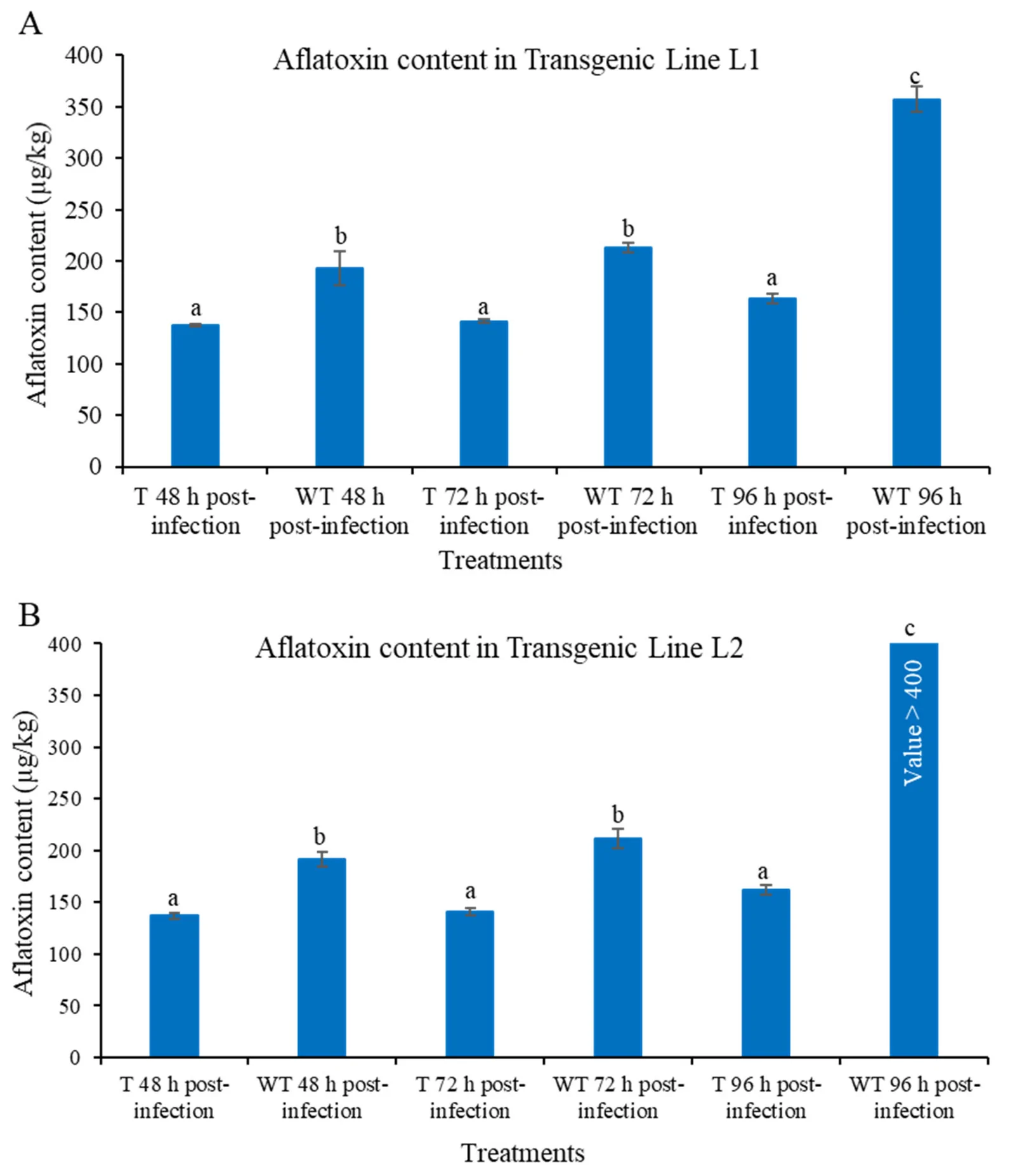
Quantification of aflatoxin B_1_ (AFB_1_) accumulation in *AhDef1* transgenic lines 1 and 2 and wild-type peanut seeds following *Aspergillus flavus* infection. (**A**) Transgenic line 1 and (**B**) transgenic line 2. Data are mean ± SE (*n* = 3), and different letters show statistically significant differences (*p*-value ≤ 0.05).

Quantification of aflatoxin B_1_ by competitive ELISA demonstrated significantly lower toxin accumulation in *AhDef1*-overexpressing peanut lines compared to wild-type plants following *A. flavus* infection (Figure 5). Across all infection time points (48, 72, and 96 h), transgenic peanut lines exhibited consistently higher B/B_0_% values compared to wild-type (WT) controls, indicating significantly lower aflatoxin accumulation following *Aspergillus flavus* infection. In line 1, based on interpolation from a four-parameter logistic (4PL) standard curve, the transgenic lines accumulated 137.7, 141.3, and 163.2 µg kg^−1^ AFB_1_ at 48, 72, and 96 h post-infection, respectively, whereas the corresponding wild-type samples accumulated 192.9, 212.8, and 357.2 µg kg^−1^, respectively. In transgenic line 2, the AFB_1_ concentration was 136.96 µg kg^−1^ at 48 h post-infection compared to 191.55 µg kg^−1^ of wild-type seeds. At 72 h, the concentrations were 140.57 µg kg^−1^ and 211.10 µg kg^−1^ in transgenic and wild-type seeds, respectively. At 96 h, the transgenic seeds showed an AFB_1_ concentration of 162.24 µg kg^−1^, whereas the corresponding wild-type sample had more than 400 µg kg^−1^ as its B/B_0_ value was below the lowest response covered by the calibration curve and therefore fell outside the validated calibration range. These results suggest that *AhDef1* expression substantially reduced aflatoxin accumulation throughout the infection period (Figure 5).

### 3.8. Polyphenol Profiling in Transgenic Peanut Lines Expressing AhDef1

Polyphenol analysis was performed using samples from two independent transgenic lines and wild-type plants. Each treatment consisted of three biological replicates, and each extract was analyzed in a technical triplicate under identical chromatographic conditions.

**Figure 6:**
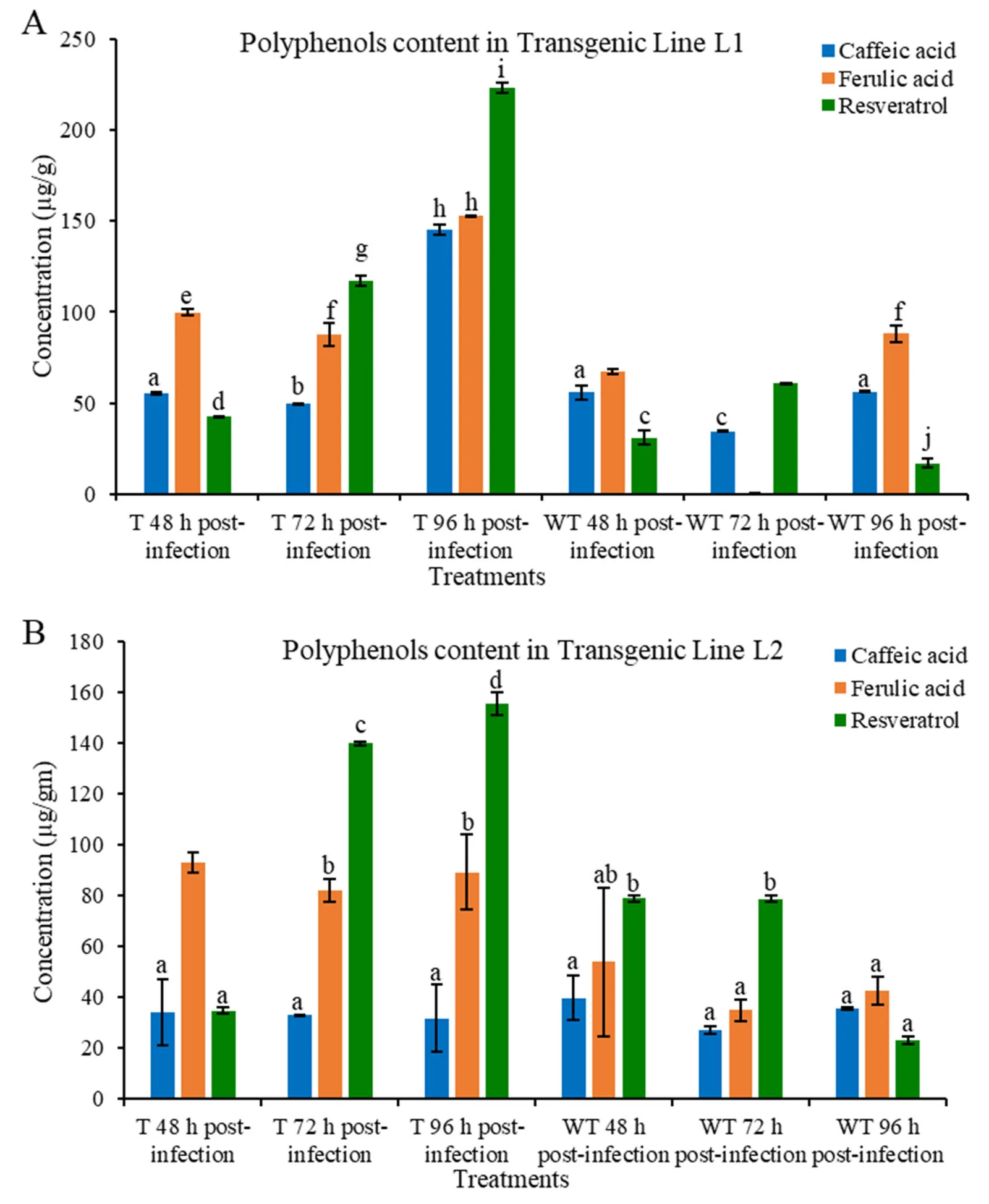
Polyphenol content in *A. flavus*-infected wild-type and transgenic peanut seeds. Quantification of polyphenol content in *AhDef1* transgenic line 1, line 2 and wild-type peanut seeds following *Aspergillus flavus* infection. (**A**) Transgenic line 1 and (**B**) transgenic line 2. Data are mean ± SE (*n* = 3), and different letters show statistically significant differences (*p*-value ≤ 0.05).

Polyphenol profiling revealed distinct accumulation patterns of caffeic acid, ferulic acid, and resveratrol across the analyzed transgenic peanut lines (Figure 6). All three polyphenols were enhanced in line 1, with the maximum accumulation found in the transgenic lines at 96 h of the infection, where resveratrol reached almost 220 µg g^−1^, followed by Ferulic acid (≈150 µg g^−1^) and caffeic acid (≈140 µg g^−1^). Transgenic line 1 and wild type showed moderate levels of polyphenol accumulation after 72 h and 48 h of infection, respectively, whereas wild type showed a significant reduction in polyphenol accumulation after 72 and 96 h of infection. This pattern suggests that phenolic metabolism is first induced in the early stages of infection and then depleted later on, most likely as a result of being used in defense-related processes.

A comparable but numerically smaller trend was noted in line 2. Resveratrol (~160 µg g^−1^) and ferulic acid (~90 µg g^−1^) once again showed the highest accumulation in transgenic line 2 after 96 h infection, although caffeic acid (~30–40 µg g^−1^) remained rather constant across all stages. Significantly, resveratrol levels were consistently higher than ferulic and caffeic acids in every sample, suggesting that *AhDef1*-expressing lines have an increased stilbene pathway flux.

### 3.9. Metabolite Profiling

More than 100 metabolites are detected, including sugars, amino acids, polyphenols, sugar alcohols, lipids, and lipid derivatives (Supplementary Table S3). GC–MS metabolomic analysis was performed using one representative single-copy transgenic line and the corresponding wild-type samples. Due to limited seed availability from the second independent transgenic line, sufficient biological material for metabolomic analysis could not be obtained. In order to examine concerted metabolic regulation during infection, Pearson correlation–based hierarchical clustering was performed for all detected metabolites at 48 h, 72 h and 96 h post-infection (Supplementary Figure S5). At 48 h, the correlation matrix exhibited two main metabolic modules with strong positive correlations between metabolites within module and negative associations of one module against another. A phenylpropanoid–lipid–sugar-related module, including ferulic acid, resveratrol, myristic acid, oleic acid, glucose and mannose were close to each other with strong positive correlations, while several organic acids and amino acids were a separated negatively associated cluster. At 72 h the network architecture was more integrated and characterized by increased intramodular connectivity. Ferulic acid and resveratrol were closely clustered with glyceric acid, glycine, valine, and fructofuranose due to the increased coordination between secondary metabolism and central carbon metabolism. Lipids generated into fatty acid products such as palmitic and stearic acids clustered together, indicating congruent fatty acid alterations.

#### 3.9.1. Ferulic Acid Fold Change (TG vs. WT)

**Figure 7:**
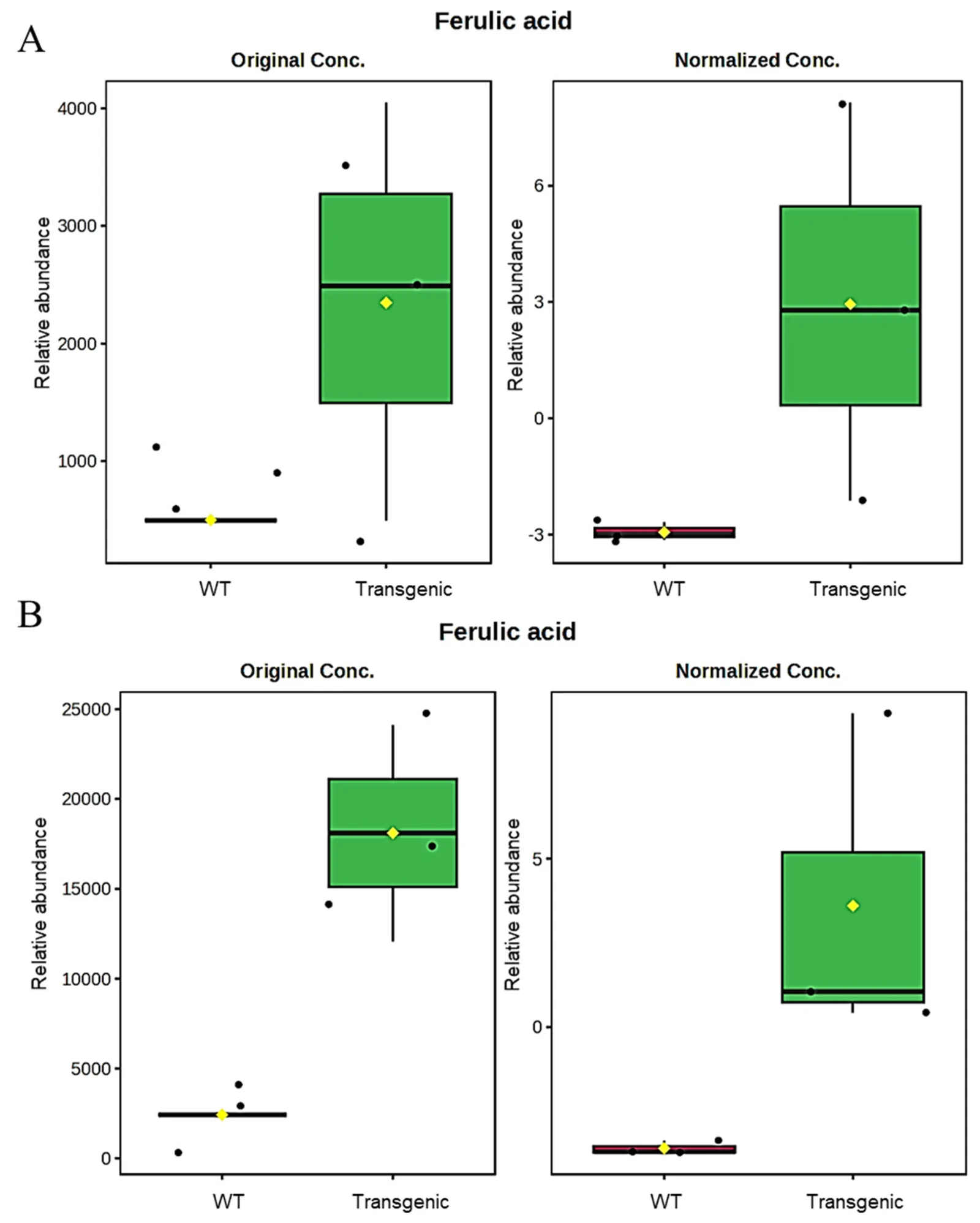
Box plot analysis of relative content of ferulic acid in transgenic peanut compared to wild type after *A. flavus* infection using the MetaboAnalyst 6.0. Box plot of relative concentrations or fold change in ferulic acid after (**A**) 72 h and (**B**) 96 h post-infection. Y-axes are represented as relative units. The bar plots show the normalized values (mean +/− SD). The yellow rhombus represents the mean concentration of the metabolite within group. Medians are indicated by horizontal lines within each box, and single data points are indicated by circles.

Ferulic acid is significantly upregulated in transgenic plants at 72 h post-infection (Figure 7). The positive normalized fold change in TG observed among phenylpropanoid-related metabolites suggests coordinated metabolic responses associated with *AhDef1* overexpression; however, these correlations do not constitute direct evidence of pathway activation. At 96 h, ferulic acid accumulation is further amplified in transgenic plants compared to WT. The sustained and enhanced upregulation suggests prolonged activation of antioxidant and cell wall reinforcement mechanisms, potentially contributing to reduced aflatoxin susceptibility.

#### 3.9.2. Resveratrol Fold Change (TG vs. WT)

**Figure 8:**
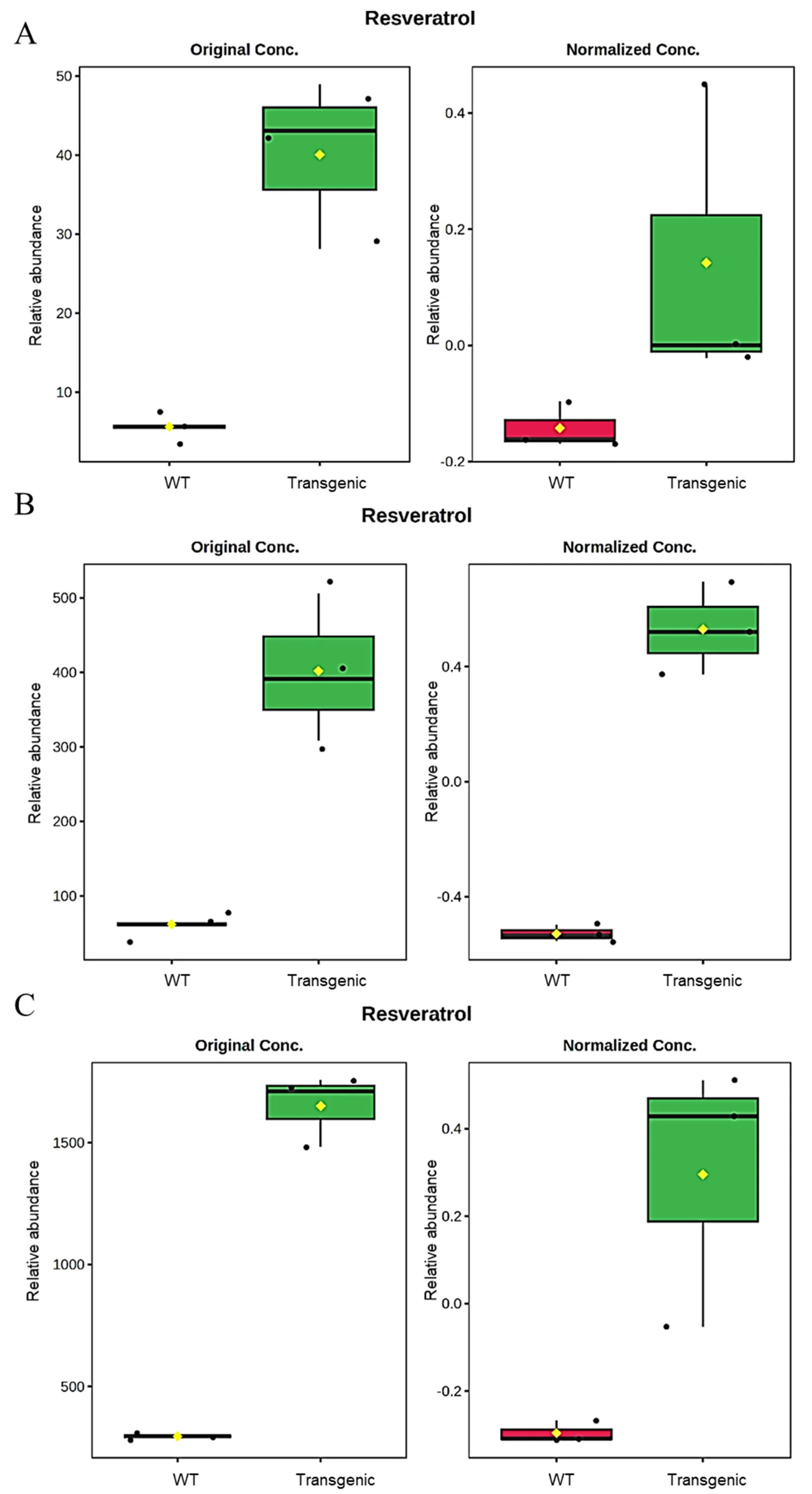
Box plot analysis of relative content of resveratrol in transgenic peanut compared to wild type after *A. flavus* infection using the MetaboAnalyst 6.0. Box plot of relative concentrations or fold change in resveratrol content after (**A**) 48 h, (**B**) 72 h and (**C**) 96 h post-infection. Y-axes are represented as relative units. The bar plots show the normalized values (mean +/− SD). The yellow rhombus represents the mean concentration of the metabolite within group. Medians are indicated by horizontal lines within each box, and single data points are indicated by circles.

Quantitative analysis revealed a strong and time-dependent induction of resveratrol in *AhDef1*-expressing transgenic (TG) lines compared to wild-type (WT) plants following *Aspergillus flavus* infection (Figure 8). At 48 h, transgenic plants exhibited a substantial increase in resveratrol accumulation relative to WT. The original concentration values showed approximately a 5–6-fold higher abundance in TG samples compared to WT controls. Normalized fold change analysis demonstrated positive enrichment in TG plants, whereas WT samples displayed negative fold change values, indicating suppression relative to baseline. At 72 h, resveratrol levels remained significantly elevated in TG plants. Although absolute concentrations were lower than at 48 h, transgenic lines consistently maintained higher levels than WT. Normalized data revealed sustained positive fold changes in TG samples, while WT plants showed negative or near-zero values. By 96 h, resveratrol accumulation in TG plants increased further, demonstrating a pronounced separation from WT samples. The original concentration data showed a several-fold enhancement in TG relative to WT. Normalized fold change values exhibited strong positive enrichment in TG plants, whereas WT samples remained negatively skewed.

## 4. Discussion

Defensins—small, cysteine-rich antimicrobial peptides—have emerged as highly promising tools for reinforcing innate immunity in peanut [23]. Plant defensins are ubiquitous in seed storage tissues and vegetative organs, where they form the first line of defense against a broad spectrum of pathogens. Structurally, they possess a conserved cysteine-stabilized αβ fold with four disulfide bridges and a γ-core motif that is critical for antifungal activity [24,25,26]. The substantial decrease in AFB1 content in transgenic seeds indicates effective inhibition of *Aspergillus flavus* colonization and subsequent toxin biosynthesis. This aligns with earlier findings that plant defensins interfere with fungal membrane integrity and pathogenesis [27,28]. Plant defensins are known to disrupt fungal membranes, interfere with ion homeostasis, and trigger intracellular stress responses in pathogens [10].

Molecular analysis is associated with single-copy integration and stable expression of *AhDef1* in transgenic lines, indicating successful genomic incorporation without complex rearrangements that could compromise transgene stability. Stable single-copy insertion is generally preferred in transgenic crops because it minimizes the risk of transgene silencing and ensures heritable expression across generations. Notably, the qRT-PCR results suggest a potential mechanism for strong pathogen-inducible expression, particularly at 48–72 h post-infection. This temporal expression profile corresponds to the early-to-mid stages of fungal colonization, suggesting that *AhDef1* participates in inducible defense responses. Similar inducible patterns have been reported for defensins in Arabidopsis and other crops, where transcriptional activation occurs rapidly following fungal challenge. In *Arabidopsis thaliana*, defensin genes such as *PDF1.2* are rapidly activated in response to fungal infection through jasmonic acid and ethylene-dependent signaling pathways, contributing to enhanced antifungal resistance [29]. The peak expression observed at 48–72 h likely represents a critical window for limiting fungal establishment. The EIA-based quantification thus provides direct biochemical evidence of the antifungal efficacy of *Defensin1* in plants [30,31]. Phenotypic infection assays demonstrated significantly delayed fungal growth in transgenic seeds compared to wild type. Importantly, ELISA-based quantification supports reduced AFB_1_ accumulation across all time points. By limiting fungal biomass and potentially altering the redox environment, defensin expression may indirectly suppress aflatoxin pathway activation. Previous studies have shown that enhancing host resistance mechanisms can significantly reduce aflatoxin contamination [32,33]. A recent study identified *AhAftr1*, a peanut aflatoxin-resistant candidate gene and validated it through transgenic experiments. Overexpression of the resistant AhAftr1 allele resulted in a 57.3% reduction in aflatoxin compared with the susceptible allele background [34].

Activation of aflatoxin biosynthesis in *Aspergillus parasiticus* contributes to alleviation of intracellular ROS stress through aflR-dependent mechanisms and regulation of antioxidant enzymes such as superoxide dismutases. Thus, interference with fungal cellular integrity and ROS balance by plant defensins may indirectly suppress aflatoxin biosynthetic activity. Since plant defensins are known to induce oxidative stress, membrane permeabilization, and apoptosis-like fungal cell death pathways, the reduced aflatoxin accumulation observed in the present study may result from combined inhibition of fungal growth and disruption of ROS-regulated toxin biosynthesis pathways [32,33]. Functional analyses of host–pathogen interactions in groundnut and maize have demonstrated that resistant genotypes activate multiple defense-associated mechanisms, including antimicrobial proteins, antioxidant enzymes, phenylpropanoid metabolism, and ROS detoxification pathways, to restrict fungal colonization and aflatoxin accumulation. Enhanced expression of antifungal proteins during the early stages of infection is considered critical for limiting pathogen establishment and toxin biosynthesis. The reduced fungal growth and lower AFB_1_ accumulation observed in *AhDef1*-expressing lines may therefore result from coordinated inhibition of fungal colonization and disruption of oxidative stress-mediated aflatoxin regulation. Previous studies have shown that oxidative stress plays a central role in activation of aflatoxin biosynthetic pathways in *A. flavus*, whereas host-derived antifungal proteins and antioxidant metabolites can interfere with fungal development and toxin production. Plant defensins are known to destabilize fungal membranes, induce ROS imbalance, and trigger fungal cell death pathways, thereby restricting pathogen progression within host tissues. These findings collectively support the hypothesis that *AhDef1*-mediated resistance enhances the intrinsic defense capacity of peanut against aflatoxigenic fungi [33].

**Figure 9:**
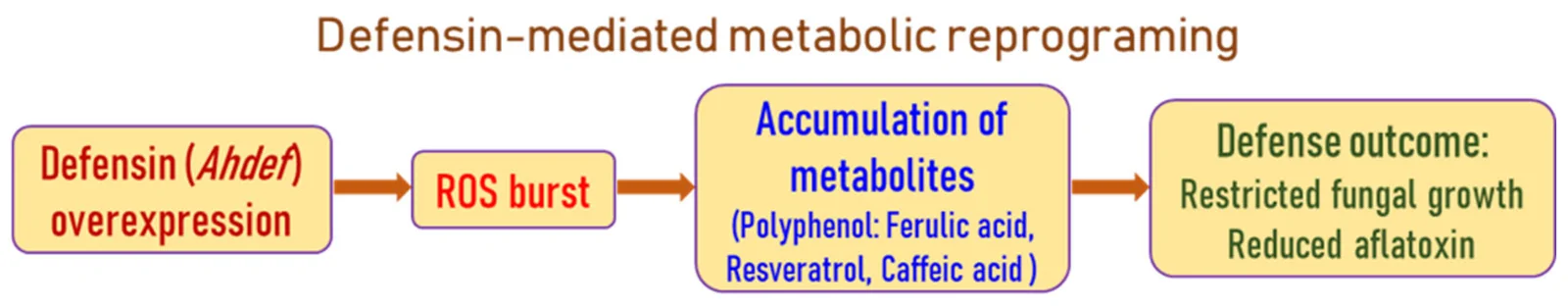
Proposed model illustrating the putative role of *AhDef1* in enhancing defense responses against *Aspergillus flavus* in peanut. The model is based on the experimental findings of the present study together with the published literature and represents a hypothetical mechanism rather than a directly validated pathway.

Metabolite profiling may contribute to extensive biochemical reprogramming in *AhDef1*-expressing peanut lines following *Aspergillus flavus* infection, indicating activation of multiple defense-associated metabolic pathways. The observed temporal shifts in metabolite abundance suggest that *AhDef1* overexpression not only confers direct antifungal activity but also modulates broader host metabolic responses during a pathogen challenge. Such large-scale metabolic reorganization is a characteristic feature of induced plant immunity, where primary metabolism is redirected toward synthesis of defense-related secondary metabolites and stress-responsive compounds. Polyphenol profiling indicates marked increases in resveratrol and ferulic acid in transgenic lines, particularly during early infection stages. Resveratrol is a well-characterized phytoalexin with strong antifungal and antioxidant activities [35,36,37]. Ferulic acid contributes to cell wall reinforcement via cross-linking of lignin and hemicellulose, enhancing structural barriers against pathogen ingress [38]. The consistently higher accumulation of resveratrol and ferulic acids suggests preferential flux through the stilbene branch of the phenylpropanoid pathway. Such redirection of metabolic flux toward secondary metabolites is a hallmark of activated plant defense. The significant fold increase in ferulic acid at 72 and 96 h post-infection further supports sustained phenylpropanoid activation. Elevated hydroxycinnamate levels enhance antioxidant capacity and contribute to reactive oxygen species (ROS) buffering during pathogen stress [14,39]. Therefore, *AhDef1* overexpression appears to reinforce both chemical and structural defense layers. These metabolomic changes suggest a potential mechanism by which *AhDef1* overexpression may enhance defense responses against *Aspergillus flavus*. The observed alterations in defense-related metabolites are associated with improved resistance; however, the underlying molecular mechanisms remain to be experimentally validated (Figure 9).

Correlation network analysis supports progressive modular strengthening from 48 to 96 h, indicating dynamic reorganization of metabolic interactions. At 48 h, initial clustering between phenolics, sugars, and fatty acids suggests early metabolic adjustment. By 72 h, enhanced integration between ferulic acid, resveratrol, amino acids, and central carbon metabolites reflects coordinated flux redistribution. At 96 h, strong modular polarization and negative correlations between primary sugars and defense metabolites indicate sustained carbon reallocation toward secondary metabolism. Such carbon partitioning from primary metabolism to defense-associated pathways has been widely documented during plant–pathogen interactions [40,41,42,43,44].

More negative correlations between the defense-associated metabolites and the primary metabolic intermediates suggest a package of metabolic flux redirections when actively defended. At 96 h, the correlation structure exhibited pronounced modular polarization, with highly robust positive correlations within the defense-related cluster. Ferulic acid showed a tight association with resveratrol, gluconolactone, citric acid, and ribofuranose, forming a highly interconnected phenolic-centered network. Concurrently, primary sugars such as glucose and sucrose demonstrated strong negative correlations with the defense module, indicating sustained carbon reallocation toward secondary metabolite biosynthesis. Based on the correlation study it was hypothesized that early metabolic adjustment had happened during initial clustering at 48 h, which was followed by strong integration that led to defense activation at 72 h, whereas metabolic reprogramming occurred at 96 h leading to protection of the plants.

The findings suggest that *AhDef1* overexpression is associated with alterations in defense-related metabolite profiles that may contribute to enhanced resistance against *Aspergillus flavus*. However, the precise roles of *AhDef1* in metabolic regulation, ROS signaling, and carbon flux remain to be elucidated through further mechanistic studies. The observed negative correlations between glucose/sucrose and phenolic compounds suggest the concept of metabolic trade-offs during immune activation. Moreover, the tight clustering of fatty acids suggests membrane remodeling and possible involvement of lipid-derived signaling molecules such as jasmonates [45]. Collectively, the strengthening intramodular correlations over time indicate stabilization of a defense-oriented metabolic state rather than a transient stress response. This system-level metabolic coordination likely underpins the reduced fungal colonization and aflatoxin accumulation observed in transgenic seeds.

Although visual assessment, aflatoxin quantification, and metabolomic analyses consistently indicated reduced fungal infection in *AhDef1*-overexpressing peanut lines, direct quantification of fungal colonization was not performed in the present study. Future studies employing fungal biomass quantification, such as quantitative PCR targeting fungal DNA or colony-forming unit (CFU) analysis, will further validate the antifungal efficacy of *AhDef1*.

From an applied perspective, the substantial reduction in fungal colonization and aflatoxin accumulation observed in *AhDef1* transgenic lines highlights the potential utility of defensin-based genetic engineering strategies for improving peanut food safety and post-harvest protection. Since aflatoxin contamination represents a major global challenge affecting crop quality, human health, and international trade, development of resistant cultivars through host-derived antimicrobial genes may provide a sustainable alternative to chemical and post-harvest control measures. Future investigations integrating transcriptomics, proteomics, and targeted pathway analysis would further clarify the regulatory networks underlying *AhDef1*-mediated resistance. In particular, characterization of jasmonic acid-, ethylene-, and ROS-associated signaling pathways, along with expression profiling of aflatoxin biosynthetic genes in *Aspergillus*, may help elucidate the precise molecular mechanisms linking defensin activity with suppression of aflatoxin production [13]. The present study has several limitations that should be considered when interpreting the findings. Although two independent single-copy *AhDef1*-overexpressing transgenic peanut lines consistently exhibited enhanced resistance to Aspergillus flavus, evaluation of additional independent transformation events and subsequent generations would further strengthen the reproducibility and stability of the observed phenotype. Furthermore, all experiments were conducted under controlled artificial inoculation conditions, and the effectiveness of *AhDef1*-mediated resistance under natural field environments remains to be established. In addition, while biochemical and GC–MS metabolomic analyses revealed significant associations between *AhDef1* overexpression and changes in defense-related metabolites, the present study did not directly investigate the underlying molecular mechanisms, including metabolic network regulation, ROS signaling, carbon flux, or fungal biomass quantification. Therefore, the proposed mechanism should be regarded as a hypothesis based on the observed molecular and metabolomic associations. Future studies integrating multiple transgenic lines, advanced generations, field validation, and functional mechanistic analyses will be essential to comprehensively establish the role of *AhDef1* in peanut defense against *Aspergillus flavus*.

## 5. Conclusions

In this study, functional characterization of the peanut defensin gene *AhDef1* demonstrated that its ectopic expression enhanced resistance to *Aspergillus flavus* infection and reduced aflatoxin B_1_ accumulation in transgenic peanut lines. *AhDef1*-overexpressing plants exhibited increased transcript abundance, reduced fungal colonization, and altered accumulation of defense-associated metabolites, including ferulic acid and resveratrol, following fungal infection. Metabolomic analysis further suggested an association between *AhDef1* expression and changes in defense-related metabolic profiles. Although the underlying molecular mechanisms require further investigation, these findings identify *AhDef1* as a promising candidate for improving fungal resistance and reducing aflatoxin contamination in peanut. Future studies involving multiple independent transgenic lines, advanced functional analyses, and field validation will further establish its potential for crop improvement. These findings provide a basis for future studies to validate the molecular mechanisms of *AhDef1*-mediated resistance and to evaluate its potential for developing aflatoxin-resistant peanut cultivars under field conditions.

## Data Availability

The original contributions presented in this study are included in the article/Supplementary Materials. Further inquiries can be directed to the corresponding author.

## Supplementary Materials

The following supporting information can be downloaded at https://www.mdpi.com/article/10.3390/cimb48090858/s1.
