# Supplementary material for: Ectopic Expression of AhDef1 Gene (Peanut Defensin Gene) Reveals Enhanced Fungal Resistance and Aflatoxin Suppression in Peanut (Arachis hypogaea L.)
Source: Curr Issues Mol Biol. 2026 Aug 24;48(9):858. doi: 10.3390/cimb48090858 (PMC13605082; doi:10.3390/cimb48090858)
Supplement: Supplementary file 1 [file cimb-48-00858-s001.zip › 01 Supplementary Table S1 and S2.pdf]

Supplementary Table S1: Primers and PCR conditions used in the study.

Event	Primer codes	Primer sequence(5'to 3')	PCR conditions
**Isolation of full length defensin-cDNA and cloning in pRT101 intermediate vector**	DEF_ F<br>DEF_R	AATCTCGAGATGGAGAAGAAAACAGTT<br>TTACCCGGGTCAACAGTTGTGAGCACA	95ºC - 3 min<br>95ºC - 25 s,<br>56ºC - 25 s,<br>72ºC - 45 s,<br>40 cycles; 72ºC - 5 min
**Defensin gene expression profiling in peanut**	DEFrt_F<br>DEFrt_R	TGGTGAAAACAGAGGCAAAGC<br>GAGCACACCAACACGCCATT	
**Confirmation of transgenic lines**	TDEF_ F<br>TDEF_R	ATGGAGAAGAAAACAGTT<br>TCAACAGTTGTGAGCACA	
	Hpt_F<br>Hpt_R	TTCTTTGCCCTCGGACGAGTG<br>ACAGCGTCTCCGACCTGATG	
**Copy number analysis**	GnVp_F<br>GnVp_R	ACACCTACAGTTGTCCCAGCTT<br>CTAGGAACCGCTTGAACCCTGT	95 ˚C - 15 s<br>60 ˚C - 15 s<br>35 cycles<br>melt curve analysis

Supplementary Table S2: Analysis of copy numbers in selected transgenic plants.

Transgenic line	Efficiency of Def RT-qPCR	Efficiency of GnVp RT-qPCR	Copy no. ratio (Def:GnVp)	Transgene copy number
1	1.030	1.032	0.9764	1
